# Eczema

**Published:** 1891-05-23

**Authors:** 


### THE PRACTITIONER'S MIRROR AND RETROSPECT.

[*The Editor will be glad to receive offers of co-operation and contributions from members of the profession. All letters should be addressed to* THE EDITOR, THE LODGE, PORCHESTER SQUARE, LONDON, W.]

### MEDICINE.

### ECZEMA.

The question how eczema should be defined is one of considerable difficulty. Is it a common superficial inflammation of the skin, or a specific disease following a definite course? If we content ourselves with the former phrase we do not mark off irritations produced artificially, and which never recur after the cause is removed, from those forms of eczema which, apparently depending on some constitutional peculiarity, recur again and again with or without the presence of an external irritant. The prognosis in the two cases, as Pye Smith points out, is as different as possible, but the pathology of the two is identical so far as we know at present.

In either, he says, there is an example of ordinary inflammation, or the reaction of living tissue to an injury, the same dilatation of vessels, slowing of blood stream, diapedesis of corpuscles, and exudation of lymph, just as we find bronchitis produced equally by irritant gases or constitutional causes. Whether, indeed, any inflammations at all occur which are not reactions to injury, but are dependent on a failure of co-ordination between nutrition and destruction, is doubtful. Certainly the number of those which cannot be traced to at least some morbid poison in the system becomes daily less. We discover, for instance, that such affections as strumous joints are dependent on bacillary irritation. If injury, then, precedes inflammation, we are driven to ask what are the internal causes that produce this state in ordinary eczema. We often hear it ascribed to the gouty or rheumatic poison or diathesis, and apparently it alternates with gouty or bronchial outbreaks. The mere presence of urate of soda in the tissues of the skin may continue, however, for a long while in eczematous subjects without any outbreak, so that no merely mechanical irritation seems likely, and in numberless cases of eczema no such poison or peculiarity in the blood can be traced. We should rather be inclined to ascribe the change to a failure of nutrition of a special type, a weakly habit of growth in the cells tending to rapid decay, as in the catarrhal affections of mucous membranes. There may be skins where the structure is such that the habit of growth is permanently feeble, and, therefore, the cells are rendered unstable by any local irritant, which should normally only stimulate their growth. Indeed, this very instability may be the cause of the deficient metabolism of gout, and in some forms of eczema the marked effect of cod liver oil and of arsenic gives weight to this view. Nothing special has been made out as to the histology of those skins which are prone to eczema. Fox, indeed, insists on their dryness. Nor, on the other hand, is there much to be said in favour of a nervous origin for the affection. Though often symmetrical, it does not usually follow the distribution of the cutaneous nerves like the kindred herpes. Still, where nerve lesions affect the nutrition of a part eczema very readily occurs.

It is noticeable that eczema constitutes a third of all skin diseases, and was originally a name for them all. Those lesions which differ in appearance have been gradually separated into other classes, and we have now left a group where the changes tend to pass through certain stages. According to Pick, these are papular, vesicular, red oozing, and scaly. In all there is much itching, and in none is there true contagiousness; but yet a tendency to spread from one spot to another, as if by a process of self-infection. The likeness in this respect between true eczemas and that caused by scabies has led many recent observers to insist on the parasitic origin of all. The fashion of referring diseases to nerve lesions and inflammation of special organs has given place to that of tracing them to the effects of micro-organisms. Now, in eczema such organisms have not been isolated, but the value of protecting threatened portions of the skin from external influences is recognised. Unna especially insists on the importance of germs as either directly producing most varieties of eczema, or causing its spread, or weakening and so predisposing the skin to the eczematous process. Thus seborrheic eczema can be best treated, he thinks, by resorcin. He applies 10 parts each of resorcin and glycerin, with 180 parts of rectified spirit, and dilutes the mixture with four times its bulk of water, soaking with this compresses of cotton wool, which he covers with oiled silk.

This view has some support from the number of germicides which rank among the most useful remedies for eczema. Thus Hutchinson, if required to choose only one remedy, would rely on tar, which, if used weak enough and freely, he says, will almost invariably cure eczema. He employs a drachm of the liquor carbonis detergens to a pint or more of water, and covers the part with rags soaked in this solution without any oiled silk, or if the eczema is very acute he employs a lotion only, adding the tar as it subsides. In short, he considers that eczema begins in an irritable skin through some local excitation, and then prospers by means of self-contagion, being generally curable by local treatment without constitutional remedies, just as he considers catarrhal affections or diphtherias may arise *de novo* from cold or damp, but soon become contagious by breeding up or affording a nidus for a special virus. In other words, an inflammation weakens the tissues, and exposes them to the attacks of parasitic germs which are always and everywhere present in our surroundings. If these cannot be kept out the eczema propagates itself. So, too, Pick lays down the rule to protect from external influence and to destroy local infection by using such applications as salicylic soap plaster and sublimate gelatine. No doubt there is an element of truth in these views. By exposure to the air an injured patch of skin is constantly affected by living irritants which depress its vitality and increase the instability of its cells, but we must not forget how easily eczema develops under aseptic dressings if they contain noxious ingredients. The primary difficulty is to diagnose the original cause of the eczema, though when once the irritable or hyper-excretive habit is set up it continues of itself or may be kept up by septic excitants. We have then to combat the septic state, the morbid habit, and the primary irritant, or we may simply endeavour to improve the nutrition of the cells so that they may conquer all three difficulties for themselves. Treatment applied to either may at times succeed. Hence the variety of means vaunted as efficacious. The primary irritant is of course easily discovered in some cases, but in many instances we are entirely in the dark. Thus, in the common eczema of the head in seemingly healthy children, is there any morbid process affecting the whole system, or is it an instability of the cells of the part accompanying the too rapid development of the hair follicles and of the soft parts beneath, a breaking down from hard work? Certainly in the absence of all symptoms we must not assume a constitutional disease, and even the efficiency of constitutional medicines instead of proving its existence, may be due merely to their alterative effect on the failing cells. Still, while we agree with Pye Smith that it is bad logic and practice to refer the difficulty to "nervous debility" and similar entities, we are at times led to believe in non-local causes, both from the analogy of the rash of scarlet fever, the effect of medicines directed to other organs, the extraordinary symmetry, and even from the hereditary character of the disease.

Its choice of the inner surfaces and folds of the limbs may be due to the fact that the protective epidermis is thinner there, and germs are less liable to be brushed away than on the outer sides, but after all there are many intense inflammations of the skin which do not go on to eczema. There is more than a mere inflammation concerned in the process. Some perversion of function like the mucoid degeneration seen in catarrhs of the mucous membranes replaces the normal activity of secretion. Frequently, as this may commence from a local injury and spread over a large area, there does seem to be a class of cases where a widely circulating poison renders unstable the cells over the entire superficies of the body, just as a single meal of bad fish induces a universal attack of urticaria.

